# Predictors of Initial Smear-Negative Active Pulmonary Tuberculosis with Acute Early Stage Lung Injury by High-Resolution Computed Tomography and Clinical Manifestations: An Auxiliary Model in Critical Patients

**DOI:** 10.1038/s41598-019-40799-w

**Published:** 2019-03-14

**Authors:** Jun-Jun Yeh

**Affiliations:** 1 Department of Pulmonary Medicine, Section of Thoracic Imaging, and Family Medicine, Ditmanson Medical Foundation, Chia-Yi Christian Hospital, Chiayi, Taiwan; 2 Chia Nan University of Pharmacy and Science, Tainan, Taiwan; 3 China Medical University, Taichung, Taiwan; 4 Pingtung Christian Hospital, Pingtung, Taiwan; 5 Heng Chun Christian Hospital, Pingtung, Taiwan

## Abstract

This study evaluated the diagnostic use of high-resolution computed tomography (HRCT), chest X-ray (CXR), and clinical manifestations (CM) to identify initial smear-negative (iSN) active pulmonary tuberculosis (aPTB) [iSN-aPTB] in patients with iSN-pulmonary diseases (PD) and acute lung injury (ALI). In the derivation cohort, the [iSN-PD] with ALI patients were divided into the [iSN-aPTB] (G1, n = 26) and [non-aPTB-PD] (G2, n = 233) groups. Lung morphology, number, and lobar (segmental) distribution were evaluated using CXR and HRCT. A multivariate analysis was performed to identify independent variables associated with G1, which were used to generate predictive score models for G1. The predictive model was validated in a separate population of patients (n = 372) with [iSN-PD] and (ALI). The validated model for [HRCT (CXR + Hypoalbuminemia)] had 93.5% (25.8%) sensitivity, 99.5% (89.4%) specificity, and a negative predictive value of 99.5% (93.0%). For [iSN-aPTB], the post-test probability in the derivation cohort (prevalence = 10%), validation cohort (prevalence = 8.3%), and the given prevalence (prevalence = 1%) was 88.7%, 94.4%, and 41.5%, respectively. The HRCT model effectively identified the [iSN-aPTB] subjects among the [iSN-PD] with ALI, regardless of CM. The [non-aPTB-PD] were also correctly classified by the HRCT and [CXR + Hypoalbuminemia] models.

## Introduction

Tuberculosis (TB) is a major health problem around the world, and early diagnosis is critical for control of TB. Although TB can affect multiple organs, it primarily involves the respiratory system. Highly contagious active pulmonary TB (aPTB) commonly presents with vague clinical symptoms such as fever, weight loss, chronic productive cough, and occasional dyspnea and hemoptysis^1^. These signs can be mistaken for community-acquired pneumonia and can be missed on the initial diagnosis in the emergency department, thereby increasing the risk of nosocomial infection and additional transmission of the disease to the general population^2^.

The acid-fast bacilli (AFB) smear of respiratory sputum is used for the rapid diagnosis of aPTB; however, this test is noted to have poor sensitivity (between 30% and 70%)^3^. The Center for Disease Control and Prevention (CDC)^4^ recommends that nucleic acid amplification (NAA), including polymerase chain reaction (PCR), should be performed on at least one respiratory specimen from each patient with signs and symptoms of pulmonary TB for whom a diagnosis of TB is being considered but has not yet been established^5^. However, the sensitivity of PCR is variable (ie, 74% to 85%) and the sensitivity for smear-negative (SN) is poor (e.g. 53% to 73%)^6^. Routine implementation of PCR on all first specimens submitted for mycobacterial culture without consideration of the clinical history is impractical and costly^4^. Results of PCR may be available within 1 day after taking a respiratory specimen; however, the rapid PCR-based assessment is dependent upon the ability to obtain a respiratory specimen. Therefore, if the initial sputum is negative (iSN), more than one day may be needed to obtain another sample and subsequent smear-positive (SP) sputum using PCR. Patients and/or family members may refuse or hesitate to receive the bronchoscopy procedure; thus, specimens cannot be obtained this way. Recent reports suggest that high-resolution computed tomography (HRCT) may be a useful tool for identifying PTB and pneumonia^7,8^, and may be performed before bronchoscopy, especially in patients with iSN^9^. If bronchoscopy cannot be performed or patients have a tendency to bleed, HRCT can be used to classify the most infectious patients (PTB) and the patients can be isolated. HRCT plays an important role in the identification of Severe Acute Respiratory Syndrome (SARS). In addition, bronchoscopy is also limited by the necessity of waiting for the results of cultures, particularly when the smear- from bronchoscopy specimens is (−, −, −).

The most common reason for admitting an aPTB patient into the intensive care unit (ICU) is the development of acute lung injury (ALI), such as acute respiratory distress syndrome (ARDS) or severe organ failure^10^. Acute respiratory failure due to aPTB often necessitates mechanical ventilation and is associated with mortality rates of approximately 14.9% to 85.5%^11,12^.

The use of chest-X-ray (CXR)^1^ and computed tomography (CT)^13^ have been investigated to determine their effectiveness in the diagnosis of aPTB in ICU patients. A case control study found that CXR was not predictive of aPTB and did not significantly contribute to the diagnosis of aPTB in the ICU^1^. One study found that the extent of disease as determined by HRCT correlated with positive smears^14^. A prior study also found that HRCT had both high sensitivity (>95.0%) and specificity (>90.0%) for predicting the presence of SP-aPTB^15^. However, the accuracy of HRCT and CXR in patients with ALI (eg, such as ARDS) for diagnosing initial smear-negative active pulmonary tuberculosis (iSN-aPTB) in early stage ALI is not well understood.

Prior studies used CXR and symptoms/signs to identify patients with SN-aPTB^16^ and aPTB^17^. The aim of the current study was to assess the diagnostic accuracy of CXR/HRCT with clinical manifestations in detecting [iSN-aPTB] and classifying [non-aPTB-PD] among patients in early stage ALI^18^ with iSN-pulmonary diseases [iSN-PD] before ICU admission or within 24 hours after ICU admission.

## Results

### Derivation cohort

#### Patient demographics and clinical characteristics

The study included patients with ALI who had 1) predicted [iSN-aPTB]: (G1; n = 26) or 2) [non-aPTB-PD] (G2; n = 233) (Table 1). The mean ages in the G1 and G2 groups were 71.38 and 70.22 years, respectively. Males comprised 53.8% and 67.3% of patients in the G1 and G2 groups, respectively. Clinical characteristics were similar between the G1 and G2 groups, including age, sex, previous TB, diabetes mellitus (DM), steroid use, chronic obstructive pulmonary disease (COPD), liver cirrhosis, uremia, upper gastrointestinal bleeding, lymphopenia, neutropenia, levels of white blood cells, and other clinical signs and symptoms. The G1 group had a significantly higher percentage of COPD and hypoalbuminemia compared with the G2 group (both P < 0.05). Patients in the G1 group also had significantly higher ALI scores, APACHE 2 scores, ante-cibum (AC) sugar, aspartate transaminase (GOT), alanine transaminase (GPT), blood urea nitrogen (BUN), and creatinine, but had lower levels of hemoglobin, albumin, PaO_2_: F_I_ O_2_, and sodium compared with the G2 group (all P < 0.05) (Table 1).

**Table 1:** Age, sex and clinical characteristics of patients with active pulmonary tuberculosis^#^ in derivation cohort. (N = 259).

Variables	^&^G1 (n = 26)	^&^G2 (n = 233)^※^	P-value
Time to PCR results in the set with the initial smear-negative and final **smear-positive result aPTB (−**, **−**, **+) n = 8; (−**, **+**, **+) n = 4; (−**, **+ −)n = 6**	3.38 ± 1.023^a^		^a^vs ^c^<0.001
**Time to aPTB culture positive in all smear negative (−**, **−**, **−) n = 8**	26.75 ± 2.91^b^		^a^vs ^b^<0.001
**Time to culture without aPTB in all smear negative (−**, **−**, **−) n = 233**		36.22 ± 10.38^c^	^b^vs ^c^ 0.001
Age*, years	71.38 ± 13.30	70.22 ± 15.60	0.716
Sex* (male [%])	14 (53.8%)	157 (67.3%)	0.192
**Underlying condition (before HRCT/CXR in early stage of acute lung injury**)^☆^			
Previous TB*	9 (34.6%)	68 (29.2%)	0.651
DM*	14 (53.8%)	93 (39.9%)	0.209
Steroid*	11 (42.3%)	56 (24.0%)	0.058
**COPD**	**15** (**57**.**7%)**^※^	**79** (**33**.**9%)**	**0**.030^*^
liver cirrhosis*	7 (26.9%)	75 (32.2%)	0.662
Uremia*	9 (34.4%)	68 (29.1%)	0.852
UGI bleeding*	7 (26.9%)	37 (15.9%)	0.170
Hypoalbuminemia <**2**.**5 g/dl**	**15** (**57**.**7%)**^※^	**51** (**21**.**9%)**	**<0**.001*
BMI <17.5 kg/(m)^2^*	15 (57.7%)	95 (40.8%)	0.142
lymphopenia*	9 (34.6%)	46 (19.7%)	0.125
neutropenia*	5 (19.2%)	51 (21.9)	0.810
**Acute condition (After CXR/HRCT in early stage of acute lung injury)**			
PaO_2:_ F_I_ O_2_<of 300	103.73 ± 57.60	145.26 ± 48.86	<0.001^*^
ALI score	2.46 ± 0.49	1.89 ± 0.38	<0.001^*^
APACHE II score	25.96 ± 6.92	19.75 ± 3.03	<0.001^*^
**Hematological study**			
Hemoglobulin, g/dL	8.83 ± 2.05	9.80 ± 1.55	0.040^*^
WBC, ×10^3^/mm^3^	10.42 ± 3.59	9.73 ± 3.36	0.322
**Biochemistry**			
AC sugar, mg/dl	249.12 ± 108.01	190.71 ± 135.52	0.034^*^
Na, mEq/L	127.23 ± 9.004	136.21 ± 8.447	<0.001^*^
**Liver function (acute hepatic failure)**			
GOT, KU/ml	122.38 ± 77.85	48.43 ± 23.58	<0.001^*^
GPT, KU/ml	101.54 ± 65.57	48.05 ± 21.58	<0.001^*^
**Renal function (acute renal failure)**			
BUN, mg/dL	94.31 ± 50.05	30.75 ± 21.38	<0.001^*^
Cr, mg/dL	3.47 ± 2.22	1.88 ± 0.79	<0.001^*^
**Symptoms and signs** ^**※**^ **(before HRCT/CXR)**			
Fever*	13 (50.0%)	72 (30.9%)	0.076
Cough > 2 weeks*	9 (34.6%)	67 (28.8%)	0.650
Dyspnea*	15 (57.7%)	106 (45.5%)	0.301
Body weight loss*	9 (34.6%)	84 (36.1%)	1.000
Weakness*	13 (50.0%)	104 (44.6%)	0.680

Data are presented as mean ± SD for continuous variables and as numbers with percentage for categorical variables.

*Statistically significant, P < 0.05. ^&^The 20/26 in G1 and 180/233 in G2 with mechanical ventilation; ^☆^Before HRCT/CXR; ^#^The diagnosis of active pulmonary tuberculosis (aPTB) based on the World Health Organization (1994) Framework for effective tuberculosis control. World Health Organization. Geneva: World Health Organization. WHO/TB/94.17. ^※^G2 including (n = 233): collagen vascular disease (n = 14), lung cancer or metastatic cancer to lung (n = 50), non-tuberculosis mycobacterium (n = 28), cryptococcosis (n = 1), pneumonia (n = 120), other lung disease (n = 10) such as pulmonary edema(n = 7) sarcoidosis (n = 1), hypersensivity pneumomnitis (n = 2).

Smear-positive aPTB (AFB+) :1) ≥2 sputum smear positive Acid Fast Bacilli (AFB+) or 2) 1 sputum smear positive for AFB Smear-positive + chest X-ray consistent with aPTB as determined by the treating medical officer or 3)1 sputum specimen positive for AFB + culture positive for AFB; Smear-negative aPTB (AFB-): 1) symptom suggestive of aPTB + ≥ 3 were negative + CXR consistent with aPTB determined by a medical official follow up by a decision to treat the patients with a full course of the anti-tuberculosis therapy or 2) diagnosis based on positive culture but negative AFB sputum.

AC, ante cibum; Cr, Creatinine, BUN, blood urea nitrogen; GOT, aspartate transaminase test; GPT, alanine transaminase test; G1, initial smear-negative active pulmonary tuberculosis (iSN-aPTB); G2 = non-aPTB pulmonary diseases [non-aPTB-PD]; DM, diabetes mellitus; WBC, white blood cells; DM, diabetes mellitus; COPD, chronic obstructive pulmonary disease; BMI, Body Mass Index (weight(kg)/height(m)^2^).

#### Imaging results

**Figure 1:**
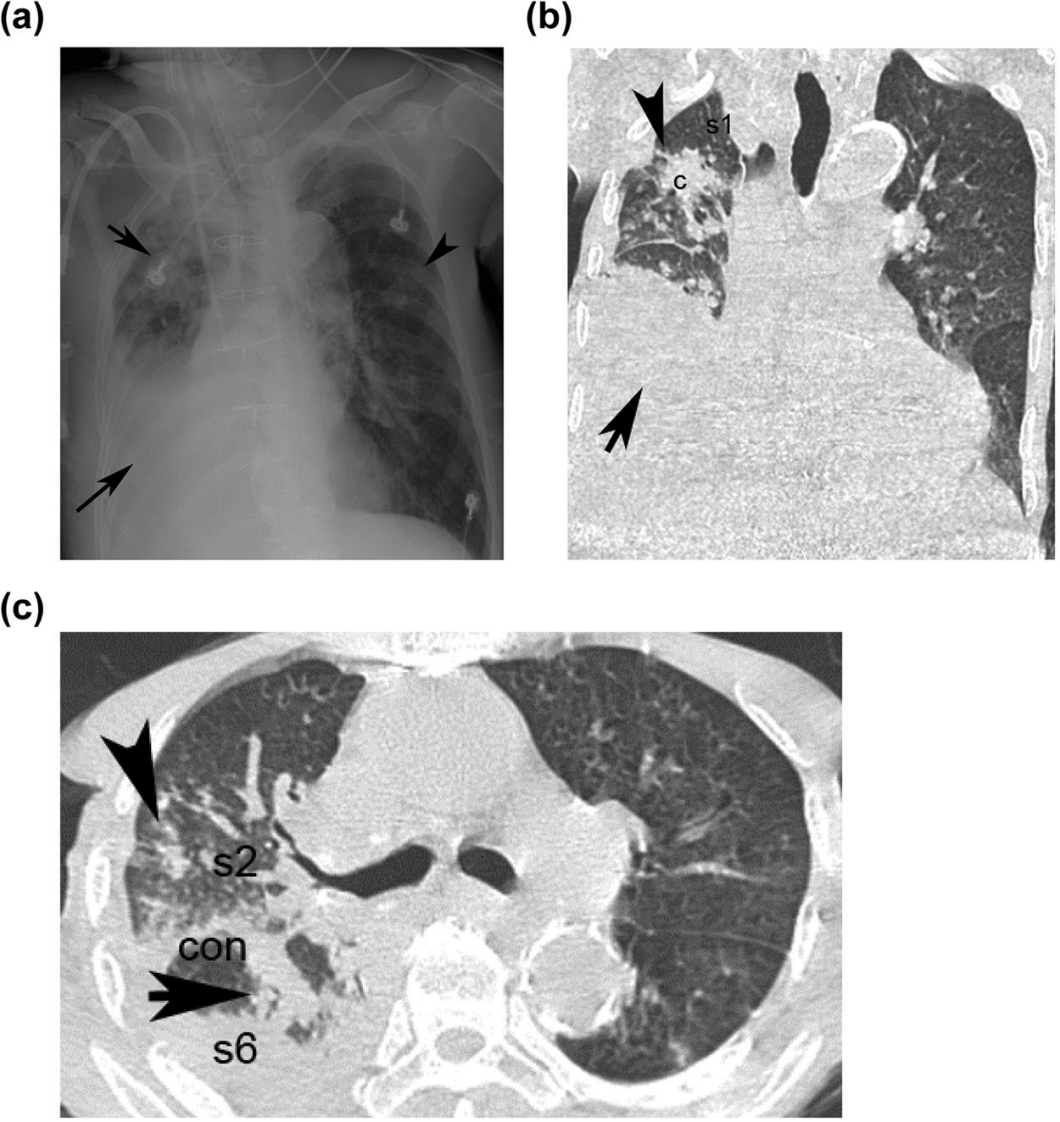
Presentation of an 82-year-male of [aPTB] with acute lung injury presenting as fever (score = 0), dyspnea (score = 0) in HRCT and [CXR + Hypoalbuminemia] model; Hypoalbuminemia (score = 1) in [CXR + Hypoalbuminemia] model. The CXR shows right (right upper black arrowhead)/left (left upper black arrow) upper lung field patch/nodules, (score = 1) and right lower lung field consolidation with pleural effusion (right lower black arrow) (score = 0) (**a**). The coronal section of HRCT shows clusters of mass of s1 of right upper lobe (right upper black arrowhead) (score = 2), pleural effusion with consolidation (right lower black arrow) of right lower lobe (**b**); The transverse section of HRCT shows clusters nodules in s2 (score = 2) of the right upper lobe (right black arrow) and consolidation in s6 of the right lower lobe (right black arrowhead) (score = 1) (**c**). The total score in [CXR (score1) + Hypoalbuminemia (score1)] model is 2; total score in the HRCT model is 3 [clusters of mass/nodules in s1/s2 of right upper lobe (score2) + consolidation in s6 of right lower lobe (score1)]. c = cluster nodules/mass; cav = cavitation; s1 = apical segment; s2 = posterior segment right upper lobe; s1 + s2 = apico-posterior segment left upper lobe; s6 = superior segment of right or left lower lobe.

**Figure 2:**
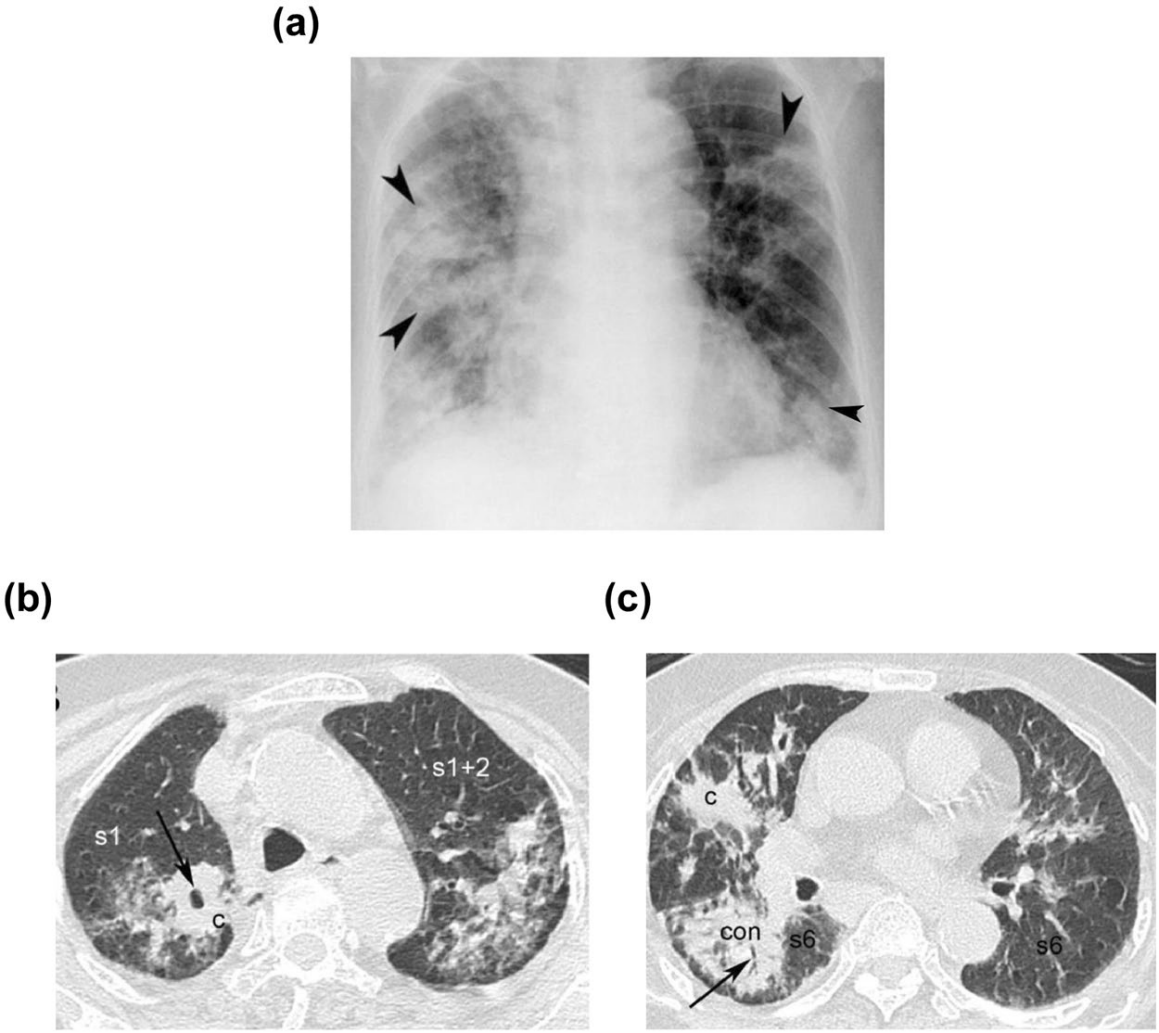
Presentation of an 84-year-male [aPTB] with acute lung injury, presenting as fever (score = 0), dyspnea (score = 0) in HRCT and [CXR + Hypoalbuminemia]; Hypoalbuminemia (score = 1) in [CXR + Hypoalbuminemia] model. The CXR shows consolidation/patch of right/left upper lung field (right/left upper black arrowhead) (score = 1), consolidation/patch of right/left lower lung field (right/left lower black arrowhead) (score = 0) (**a**). The transverse section of HRCT shows cluster nodules/mass (black arrow) (score = 2) with cavitation (score = 0) in the s1 of right upper lobe, (**b**) consolidation (black arrow) in the s6 of the right lower lobe (score = 1). (**c**) The total score in the [CXR (score1) + Hypoalbuminemia (score1)] model is 2, the total score in the HRCT model is 3 [cluster nodules/mass (score = 2) with cavitation (score = 0) in the s1 + consolidation in the s6 of the right lower lobe (score = 1)]. (**c**) cluster nodules/mass. con:consolidation; s1 = apical segment; s2 = posterior segment right upper lobe; s1 + s2 = apico-posterior segment left upper lobe; s6 = superior segment of right or left lower lobe.

CXR results indicated that the frequency of the consolidation/patch/nodules of the right/left upper lung field were higher in G1 patients than in G2 patients (73.1% vs. 38.6%, P value < 0.001; Figs 1 and 2). Cavitation of the right/left upper or lower lung field was also more prevalent in G1 patients than in G2 patients (upper: 23.1% vs. 1.3%, P < 0.001; lower: 26.9% vs. 2.6%, P < 0.001) (Table 2).

**Table 2:** HRCT morphology, anatomy distribution and CXR findings in derivation cohort. (N = 259).

		G1 (n = 26)	G2 (n = 233)	P-value
***HRCT morphology*** ^※^				
Consolidation		25 (96.2%)	163 (70.2%)	0.002^*^
RUL/LUL^★^	s1, s2, s1 + s2, s3	14 (53.8%)	109 (46.8%)	0.539
RML/LingL^★^	s4, s5	12 (46.2%)	141 (60.5%)	0.207
Superior segment of RLL/LLL	s6	24 (92.3%)^※^	14 (6.0%)	<0.001^*^
Other segments of RLL/LLL^★^	s7, s8, s7 + 8, s9, s10	11 (42.3%)	102 (43.8%)	1.000
Cavitation		12 (46.2%)	16 (6.90%)	<0.001*
RUL/LUL^★^	s1, s2, s1 + s2, s3	11 (42.3%)	2 (0.9%)	<0.001^*^
RML/LingL^★^	s4, s5	5 (19.2%)	14 (6.0%)	0.030^*^
Superior segment of RLL/LLL^★^	s6	6 (23.1%)	3 (1.3%)	0.001^*^
Other ^&^segments of RLL/LLL^★^	s7, s8, s7 + 8, s9, s10	6 (23.1%)	17 (7.3%)	0.018^*^
Clusters nodules/mass		25 (96.2%)	9 (3.9%)	<0.001^*^
RUL/LUL	s1, s2, s1 + s2, s3	25 (96.2%)^※^	8 (3.4%)	<0.001^*^
RML/LingL^★^	s4, s5	4 (15.4%)	6 (2.6%)	0.011^*^
Superior segment of RLL/LLL^★^	s6	9 (34.6%)	0 (0.0%)	<0.001^*^
Other segments of RLL/LLL^★^	s7, s8, s7 + 8, s9, s10	10 (38.5%)	11 (4.7%)	<0.001^*^
Tree-in-bud^★^		20 (76.9%)	166 (71.2%)	0.650
Centri-lobular nodules^★^		24 (92.3%)	209 (89.7%)	1.000
Military nodules/reticular nodules^★^		11 (42.3%)	8 (3.4%)	<0.001^*^
Bronchial thickening^★^		22 (84.6%)	175 (75.1%)	0.341
Interlobular thickening^★^		22 (84.6%)	204 (87.6%)	0.755
Ground-glass opacity^★^		25 (96.2%)	188 (80.6%)	0.057
Pleural effusion^★^		9 (34.6%)	36 (15.5%)	0.025*
***CXR***				
**Consolidation/patch/nodules**				
Right/left upper lung field		19 (73.1%)^※^	90 (38.6%)	<0.001^*^
Right/left lower lung field^★^		21 (80.8%)	211 (90.6%)	0.165
**Cavitation**				
Right/left upper lung field^★^		6 (23.1%)	3 (1.3%)	<0.001^*^
Right/left lower lung field^★^		7 (26.9%)	6 (2.6%)	<0.001^*^
Consolidation number ≧3^★^		23 (88.5%)	180 (77.3%)	0.220
Miliary nodules/reticular nodules^★^		8 (30.8%)	4 (1.7%)	<0.001*

Data are presented as numbers with percentage. *Statistically significant, P < 0.05.

^※^Indication of HRCT (n = 259): 1) suspected acute pneumonia/mycobacterium infection with equivocal finding in CXR (n = 60) or suspected complicated pneumonia/mycobacterium infection in CXR (n = 69), interstitial lung disease, (n = 41) lung cancer or cancer with *metastasis* to lung (with small patch or nodules in CXR) (n = 66) or occult lung disease with equivocal finding in CXR such as bullae or minimal pneumothorax (n = 23).

CXR, chest X-ray; G1, initial smear-negative active pulmonary tuberculosis (iSN-aPTB); G2 = non-aPTB pulmonary diseases [non-aPTB-PD]; HRCT, high-resolution computed tomography; LLL, left lower lobe; LUL, left upper lobe; LingL, Left lingual lobe; RLL, right lower lobe; RML, right middle lobe; RUL, right upper lobe; s1 = apical segment; s2 = posterior segment right upper lobe; s1 + s2 = apico-posterior segment left upper lobe; s3 = anterior segment; s4 = lateral segment of right middle lobe or super segment of left lingual lobe; s5 = medial segment of right middle lobe or inferior segment of left lingual lobe; s6 = superior segment of right or left lower lobe; s7 = medial segment of right lower lobe; s8 = anterior segment of left lower lobe; s7 + s8 = medial-anterior segment of left lower lobe; s9 = lateral segment of right or left basal lower lobe; s10 = posterior segment of right or left basal lower lobe. RUL: s1 + s2 + s3; LUL: s1 + 2 + s3; RML: s 4 + s5, LingL: Ls4 + s5; s6: superior segment of right or left lower lobe (RLL/LLL).

Among the HRCT morphology findings, the frequency of consolidation in the superior segment (s6) of the right lower lobe/left lower lobe [RLL/LLL] was greater in G1 patients than in G2 patients (92.3% vs. 6.0%, P < 0.001) (Table 2). The frequency of clusters of nodules/mass or cavitation in all regions of the lung analyzed was greater in G1 patients compared with G2 patients (all P ≤ 0.030). The frequency of military/reticular nodules (42.3% vs. 3.4%, P < 0.001) and pleural effusion was greater in G1 patients than in G2 patients (34.6% vs. 15.5%, P = 0.025) (Table 2).

### Multivariate analysis of predictors for diagnosing PTB

Multivariate analysis was performed to identify independent variables that may be predictive of [iSN-aPTB], including variables identified in the [underlying disease + symptom/sign + CXR] or [underlying disease + symptoms/signs + HRCT] that were significantly different between groups (*P < 0.05); statistically significant (P < 0.05) and percentage >50% in G1 or G2 (Tables 1 and 2). Analysis using findings from the [underlying disease + symptoms/signs + CXR] found that [consolidation/patch/nodules of the right/left upper lung field, Hypoalbuminemia] were independent variables for [iSN-aPTB] (OR = 9.13, 10.207, both P < 0.05; [Hypoalbuminemia + CXR] model) (Table 3).

**Table 3:** Summary of the results of ^#^multivariate logistic regression identifying subjects with active PTB associated with multiple predictors in the derivation cohort. (N = 259).

Term	Estimated β (SE)	Estimated Odds Ratio [95% CI]	*P*-value	Relative Score^a^
***HRCT model*** **(variables: COPD**, **Hypoalbuminemia**, **Cluster nodules/mass s1**, **s2**, **s1 + 2**, **S3**, **Consolidation s6)**				
**Cluster nodules/mass s1**, **s2**, **s1 + 2**, **S3 (RUL/LUL)**	5.376 (1.39)	216.174 [14.168–3298.488]	0.000^*^	2
**Consolidation s6**	3.006 (0.969)	21.977 [2.2382–202.74]	0.002^*^	1
*after* ^#^multivariate logistic regression, the variables (e.g. COPD, Hypoalbuminemia) with P values > 0.05, the relative score of these 2 variables set as 0				
***CXR*** ** + Hypoalbuminemia model (variables: COPD, Hypoalbuminemia, Consolidation/patch/nodules RUL/LUL)**				
**Hypoalbuminemia**	2.323 (0.509)	10.207 [3.761–27.7]	<0.001	1
**Consolidation/patch/nodules** **RUL/LUL**	2.212 (0.542)	9.13 [3.15–26.40]	<0.001^*^	1
*afte r*^#^multivariate logistic regression, the variable (e.g. COPD) with P value > 0.05, the relative score of this variable set as 0				

^&^Other variables of the *P value > 0.05 or *frequency < 50% in Tables 1 and 2 (before HRCT/CXR). The relative score of these variables set as 0 in [HRCT] model and [CXR + Hypoalbuminemia] model.

*Statistically significant (p < 0.05). SE, standard error.

^#^Only the P value < 0.05 and the frequency > 50% in the symptom/sign, underlying disease of Table 1 (^☆^before patients received the HRCT and CXR) and finding of HRCT(CXR) of Table 2 (e.g. COPD, Hypoalbuminemia, cluster nodules/mass s1, s2, s1 + 2, s3 Consolidation s6 in HRCT, consolidation/patch/nodules in CXR) were enrolled into the multivariate logistic regression.

^a^After the multivariate logistic regression only the variable keep the P value < 0.05 (cluster nodules/mass s1, s2, s1 + 2, s3 and consolidation s6) in HRCT model; (consolidation/patch/nodules and Hypoalbuminemia) in [CXR + Hypoalbuminemia] model were enrolled into the score system. In the [HRCT] model, the relative score is given as 1, if β value > 0 < 5 and the relative score is set as 2, if β value ≥ 5. In the [CXR + Hypoalbuminemia] model, the relative score is given as 1, if β value greater than 0 and less than 5.

RUL: s1 + s2 + s3; LUL: s1 + 2 + s3; RML: s4 + s5, LingL: Ls4 + s5; s6: superior segment of right or left lower lobe (RLL/LLL).

^&^Before the multivariate logistic regression, the *P value > 0.05 such as variable of symptom/sign in Table 1 (e.g. age, sex, fever, cough for >2 weeks, dyspnea, body weight loss, weakness) or HRCT (e.g. consolidation of any segment of both lobes except of the s6, tree-in-bud, centrilobular nodules, bronchial thickening, ground-glass opacity) or CXR morphology (e.g., consolidation/patch/nodules of right/left lower lung field, consolidation≥3) or the [*frequency < 50%] such as the variables of HRCT (e.g. cavitation of any segment of both lobes, clusters nodules/mass of any segment of both lobes except of the right/left upper lobe, pleural effusion) or CXR morphology (e.g. cavitation of right/left upper or lower lung field, military nodules) in the Table 2 were also set the relative score as 0.

CXR, chest X-ray; G1, iSN-aPTB; G2, [non-aPTB-PD]; HRCT, high-resolution computed tomography; s1 = apical segment; s2 = posterior segment right upper lobe; s1 + s2 = apico-posterior segment left upper lobe; s3 = anterior segment; s6 = superior segment of right or left lower lobe.

G1, initial smear-negative active pulmonary tuberculosis (iSN-aPTB); G2 = non-aPTB pulmonary diseases [non-aPTB-PD].

Multivariate analysis of the [underlying disease + symptoms/signs + HRCT] indicated that [cluster nodules/mass of the RUL/LUL, consolidation in the s6 of RLL/LLL] were independent variables for [iSN-aPTB] (OR = 216.174, 21.977; both P < 0. 05: [HRCT] model) (Table 3).

### Diagnostic model and evaluation of diagnostic accuracy (to set relative score)

Prior to the multivariate analysis, the *P value > 0.05 of variable of symptoms/signs in Table 1 (e.g., age, sex, fever, cough for >2 weeks, dyspnea, body weight loss, weakness) or HRCT (e.g., consolidation of any segment of both lobes except s6, tree-in-bud, centrilobular nodules, bronchial thickening, ground-glass opacity) or CXR morphology (e.g., consolidation/patch/nodules of right/left lower lung field, consolidation ≥3) or the [*frequency <50%] for variables of HRCT (e.g., cavitation of any segment of both lobes, clusters nodules/mass of any segment of both lobes except of the right/left upper lobe, pleural effusion) or CXR morphology (e.g., cavitation of right/left upper or lower lung field, military nodules) in Table 2 were also set with a relative score of 0 (Table 2).

Multivariate analysis found that for the [Hypoalbuminemia + CXR] model, the estimated β value of the consolidation/patch/nodules of the right/left upper lung field was 2.21 and for hypoalbuminemia was 2.32. Since the estimated β values were greater than 0 and less than 5, the relative score was set as 1 (Table 3). Multivariate analysis for the [HRCT model] found the estimated β value for consolidation of the s6 of RLL/LLL (upper/middle lung field) was greater than 0 and less than 5 (the estimated β = 3.006), and hence. the relative score was set as 1 (Table 3). The relative score for RUL/LUL of cluster nodules/mass (upper/middle lung field) was set as 2; as the estimated β value was greater than 5 (the estimated β value = 5.376) (Table 3). The lesion in the upper/middle lung field was only rarely affected by the respiratory effect. Other variables in the [Hypoalbuminemia + CXR] model (variables: COPD, P value > 0.05), or [HRCT] model (variables: COPD, hypoalbuminemia, P value > 0.05) were not revealed and were set as 0 (Table 3). Other variables in the [underlying disease + symptom/sign + CXR] or [underlying disease + symptom/sign + HRCT] models were not revealed and were set as 0 (Table 3).

The current model differs from the model in our prior study^19^ in at least 4 ways: 1) We did not include clinical manifestation in the analysis of the previous model^19^; 2) In the present study, we set the β value > 0 and <5 with a relative score of 1 and an estimated β value > 5 (the estimated β value = 5.376) was given a relative score of 2; 3) In the prior study, we divided the larger estimated β value by the smallest estimated β value to obtain the relative score^20^; 4) The scoring of the present study is simpler and easier to use, and was not confounded by the use of bronchiectasis as a predictor, which may be due to the artifact of pseudo-bronchiectasis. Moreover, in the present study, owing to that fact that ICU is a critical source for severe nosocomial infection, we enrolled patients with (−, +, −), (−. −. + ), (−, −, −), or (−, + , + ) smear results into the iSN-aPTB group. However, only patients with (−, +, −), (−, −, +), or (−, + , + ) smear findings were included in the previous study iSN-aPTB group^19^.

**Figure 3:**
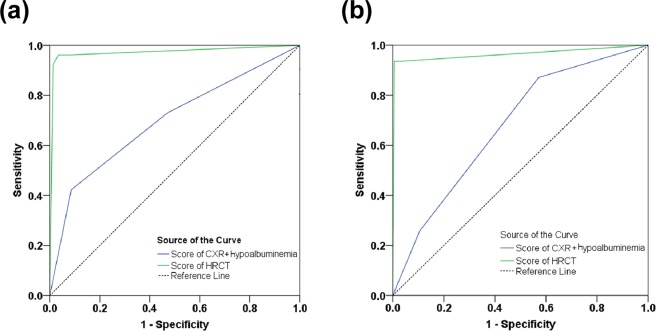
Area under curve (AUC) of the CXR model. The Receiver operator characteristic (ROC) curve of the derivation cohort (**a**) and the validation cohort (**b**). The area under the ROC curve was derived as 0.698 (95% CI = 0.579 to 0.817) for score of score of [CXR + Hypoalbuminemia] model and 0.972 (95% CI = 0.927 to 1.000) for score of HRCT model in derivation cohort. They are under of the ROC curve was derived as 0.677 (95% CI = 0.586 to 0.769) for score of [hypoalbuminemia + CXR] model and 0.964 (95% CI = 0.913 to 1.000) for Score of HRCT model.

Receiver operator characteristic (ROC) curves for the [CXR + Hypoalbuminemia] and HRCT are shown in Fig. 3. For the CXR model, the area under the curve (AUC) in the derivation cohort [validation cohort] was 0.698 ± 0.061 (95% CI = 0.579–0.817) [0.677 ± 0.047 (95% CI = 0.586–0.769)] (both P values < 0.001). With an ideal cut-off point score of 2, the sensitivity and specificity for identifying [iSN-aPTB] by [CXR + Hypoalbuminemia] was 42.3% and 91.4%, respectively, in the derivation cohort (Table 4) and was 25.8% and 89.4% in the validation cohort (Table 5). Positive predictive value (PPV) and negative predictive value for [CXR + Hypoalbuminemia] was 35.5% and 93.4%, respectively, in the derivation cohort (Tables 4), and 23.5% and 93.0% in the validation cohort (Table 5).

**Table 4:** Sensitivity, specificity, positive predictive value, and negative predictive value of HRCT and [CXR + Hypoalbuminemia] models in the derivation cohort. (N = 259).

Predictive results	[CXR + Hypoalbuminemia] model		HRCT model	
	Predicted iSN-aPTB (n = 31) ≥2	Predicted [non-aPTB-PD] (n = 228) <2	Predicted iSN-aPTB (n = 27)≥3	Predicted [non-aPTB-PD] (n = 232) <3
G1 (n = 26)	11	15	24	2
G2 (n = 233)	20	213	3	230
**Predictive terms**				
Sensitivity	42.3% (11/26)		92.3% (24/26)	
Specificity	91.4% (213/233)		98.7% (230/233)	
False positive rate	8.6% (20/233)		1.3% (3/233)	
False negative rate	57.7% (15/26)		7.7% (2/26)	
Positive predictive value	35.5% (11/31)		88.9% (24/27)	
Negative predictive value	93.4% (213/228)		99.1% (230/232)	

CXR, chest X-ray; False positive rate = 1-specificity; False negative rate = 1-sensitivity.

The cut-off value were 2 in the [CXR + Hypoalbuminemia] model and 3 in the HRCT model from the predictive score classified patients as G1, iSN-aPTB; (> = 2 in the [CXR + Hypoalbuminemia] model and >= 3 in the HRCT model) or G2 = Non-aPTB-pulmonary diseases [non-aPTB-PD] (<2 in the [CXR + Hypoalbuminemia] model and < 3 in the HRCT model).

Derivation cohort prevalence = 26/259 = 10.0%; pre-test odd ratio 10.0/[100.0 − 10.0] = 0.11, likelihood ratio = 92.3/1.3 = 71, post-test odd ratio = 0.11 × 71 = 7.81, post-test probability = 7.81/[7.81 + 1.00] = 88.7%.

Given prevalence = 1%, 1/[100 − 1] = 0.01, 0.01 × 71 = 0. 71, 0.71/[1.00 + 0.71] = 41.5%.

G1, initial smear-negative active pulmonary tuberculosis (iSN-aPTB); G2 = non-aPTB pulmonary diseases [non-aPTB-PD].

**Table 5:** Sensitivity, specificity, positive predictive value, and negative predictive value of HRCT and CXR plus Hypoalbuminemia models in the validation cohort^※^ (N = 372).

Predictive results	CXR + Hypoalbuminemia score		HRCT score§	
	Predicted iSN-aPTB (n = 44)	Predicted [non-aPTB-PD] (n = 328)	Predicted iSN-aPTB (n = 31)	Predicted [non-aPTB-PD] (n = 341)
	≥2	<2	≥3	<3
G3 (n = 31)	8	23	29	2
G4 (n = 341)	36	305	2	339
**Predictive terms**				
Sensitivity	25.8% (8/31)		93.5% (29/31)	
Specificity	89.4% (305/341)		99.5% (339/341)	
False positive rate	10.6% (36/341)		0.5% (2/341)	
False negative rate	74.2% (23/31)		6.5% (2/31)	
Positive predictive value	23.5% (8/44)		93.5% (29/31)	
Negative predictive value	93.0% (305/328)		99.5% (339/341)	

CXR, chest X-ray; False positive rate = 1-specificity; False negative rate = 1-sensitivity. G3, initial smear-negative active pulmonary tuberculosis (iSN-aPTB); G4 = non-aPTB pulmonary diseases [non-aPTB-PD].

^※^G4 including (n = 341): collagen vascular disease (n = 10), lung cancer or metastatic cancer to lung (n = 18),smear-negative aPTB (n = 20),non-tuberculosis mycobacterium (n = 15), cryptococcosis (n = 1), aspergillosis (n = 2), pneumonia (n = 241), actinomycosis (n = 1), other lung disease (n = 33) such as pulmonary edema(n = 25) sarcoidosis (n = 1), hypersensivity pneumomnitis (n = 7).

Validation cohort prevalence = 31/372 = 8.3%; pre-test odd ratio 8.3/[100 − 8.3] = 0.091; likelihood ratio = 93.5/0.5 = 187; post-test odd ratio = 0.091 × 187 = 17.02; post-test probability = 17.02/[17.02 + 1] = 94.4%.

^§^We isolated these patients and try the anti-TB therapeutic drug, if the patients have the HRCT total score ≥ 3. After the confirmed diagnosis of these 372 patients, we test the ability of this model in the validation cohort.

The AUC value under the ROC curve for the HRCT model in the derivation cohort [validation cohort] was 0.972 ± 0.023 (95% CI = 0.927–1.000) [0.964 ± 0.026 (95% CI = 0.913–1.000)] (both P < 0.001). With an ideal cut-off point score of 3, the sensitivity and specificity for identifying [iSN-aPTB] with HRCT was 92.3% and 98.7%, respectively, for the derivation cohort, and 93.5% and 99.5% for the validation cohort (Tables 4 and 5). Positive predictive value and NPV for the derivation cohort were 88.9% and 99.1%, respectively, and for the validation cohort were 93.5% and 99.5% (Tables 4 and 5). The post-test probability in the derivation cohort (prevalence = 10%), validation cohort (prevalence = 8.3%) and given prevalence (prevalence = 1%) was 88.7%, 94.4%, and 41.5%; respectively. The relative score of CXR (including Radiologist 1, Radiologist 2, and Radiologist 3), HRCT, and [CXR + Hypoalbuminemia] are summarized in Supplementary Table S1, and the distribution of patients’ scores with Radiologist 1 vs. Radiologist 2 for HRCT and CXR in the derivation cohort is presented in Supplementary Table S2. The actual timing of artery blood gas (P/F ratio < 300 mmHg), bilateral infiltration in Chest-x-ray and HRCT was showed in Supplementary Table S5.

## Discussion

Early and prompt diagnosis of [iSN-aPTB] is important for treating affected patients and inhibiting spread of the disease. The diverse clinical presentation of patients with [iSN-aPTB] and possibly limited healthcare resources (particularly during an influenza outbreak) may complicate effective management of [iSN-aPTB]. The present study was designed to identify independent disease-related variables associated with symptoms/signs, CXR and HRCT of [iSN-aPTB] and to determine the diagnostic accuracy of models that incorporated these variables in diagnosis [iSN-aPTB] in patients with early stage ALI. Using the HRCT model, we were able to identify patients with [iSN-aPTB] among the [iSN-PD] patients with ALI in the validation cohort, regardless of the clinical manifestations. Furthermore, the highly predictive negative values were found in the [CXR + Hypoalbuminemia] (93.0%; in validation cohort) and the HRCT (99.5%; in validation cohort) model. Therefore, using these two models, it was possible to identify patients with [non-aPTB-PD] among [iSN-PD] patients with scores <2 (305/328) in the [CXR + Hypoalbuminemia] model and scores <3 (339/341) in the HRCT model.

Multivariate analysis identified independent variables for [CXR + Hypoalbuminemia] and HRCT model associated with [iSN-aPTB]. Based on these independent variables, a predictive score for [iSN-aPTB] was generated to differentiate G1 (G3) and G2 (G4) patients. ROC analysis found that the HRCT had great diagnostic accuracy in identifying patients with [iSN-aPTB]. Validation of the models developed when the derivation cohort of the study found that HRCT had high sensitivity (93.5%).

Higher sensitivity may help clinicians to evaluate the therapeutic response of the anti-tuberculosis drug within 24 hours. These findings indicate that HRCT has good diagnostic accuracy for identifying [iSN-aPTB] in ICU patients with ALI. The benefit of HRCT over smear and culture testing for AFB among ICU patients with [iSN-aPTB] is the fact the results are available within a single day. Because HRCT allows the initiation of anti-TB treatment before culture or PCR results are available, it is especially useful in patients with iSN, consistent with our previous study^19^.

Although the current model does not replace the need for culture evaluation or the PCR results, diagnosis of [iSN-aPTB] is often delayed while waiting for culture results and diagnosis is often made based on the [CXR + clinical response]^20^, resulting in empirical anti-TB treatment without microbiological confirmation^21^. Results of this study highlight the HRCT model, demonstrating that it is a useful method in patients having ALI without significant differences in the symptoms/signs between the [iSN-aPTB] and the [non-aPTB-PD], or respiratory specimens are unavailable and CXR reveals atypical presentation^22^. Thus, this model may help clinicians to try anti-TB drugs and monitor the therapeutic response for the patients with scores ≥3 while waiting for the final TB culture or the PCR results, if the initial sputum assessments were negative.

A recent study found that concomitant bacterial and PTB infections are not uncommon, with a frequency of about (6/39, 15%)^23^. In the present study, we excluded patients with concurrent bacteria plus PTB in the derivation (n = 4/54) and validation cohorts (n = 6/161) during the iSP phase. These 10 patients received antibiotics prior to receiving AFB results. After the AFB results were known, the patients were treated with anti-TB and antibiotics if a bacterial aPTB was present. Patients in the derivation (n = 120) or validation (n = 241) cohort with a bacterial infection were treated with antibiotics.

The CT angiogram sign of consolidation^24^ may help to differentiate malignant lesions from benign lesions; however, in the present study, enhancement of the consolidation lesion with contrast medium was not routinely owing to the poor renal function typical of ALI (Table 1). Even the benign lesions such as PTB in the s6 of the RLL/LLL have higher frequency than malignant lesions; the malignant lesion may present as consolidation in the s6 of the RLL/LLL (score = 1), which may confound the results. Finally, using different milliamperage for settings has not been performed previously^25^. Therefore, image quality scores^26^ for repeat scans^27^ obtained with these 2 cohorts were unavailable, owing to the critical condition of the patients. We used the interobserver total score in replace of the Image Quality Scores, which showed good results (all kappa values > 0.8). In these models, only the upper/middle lung field, including the [hypoalbuminemia + CXR- RUL/LUL consolidation/patch/nodules] model and the [HRCT] model (the RUL/LUL of cluster nodules/mass with s6 consolidation of RLL/LLL), were used with the idea of attenuating the confounding factors of cardiac/respiratory artifacts. However, whether or not to use the Image Quality Score in the ALI patients warrants further investigation.

Severe aPTB in ICU is a critical source of nosocomial infection. CT chest scans may help in gathering diagnostic information of the ALI patients in whom TB are suspected but not confirmed, and will also allow for targeting using bronchoscopy or CT-guided biopsy^28,29^ Through 2012 January to 2012 July the new validation model [iSN-aPTB with concomitant bacteria] had the likelihood of up to 130 based on the HRCT score (Supplementary Table S3). Giving the prevalence 0.1%, the post-test probability was 56.5 > 5%, which is the optimal level of cut-off threshold for isolation, as described previously^16^. Therefore, the model has the potential for identifying the iSN cohort in the high to lower prevalence range. Nevertheless, this result warrant additional study.

Several limitations should be considered when interpreting the findings of the present study. First, our analysis was limited to the variables we evaluated using the [CXR + Hypoalbuminemia] and HRCT models. It is possible that other clinical variables may also be predictive of [iSN-aPTB]. In addition, our patient population was drawn from a limited geographical region and it is not known how our findings may translate to other regions. Additional studies are required from other countries and populations to further validate our models. In order for patients to receive HRCT after invasive mechanical ventilation (n = 5 in derivation cohort, n = 6 in validation cohort), it is necessary for a physician to be on call/available. Usually, in Taiwan hospitals, the emergency room (ER) is close to the CT room so the transport time is within 10 minutes for nearly all patients^30^. The ER is staffed by experienced emergency doctors who are on duty 24 hours a day. Meanwhile, a large portion of patients in the derivation (254/259) and validation (366/372) cohorts received HRCT at ER without invasive mechanical ventilation.Only (5/259) and (6/372) received HRCT after the invasive mechanical ventilation during ICU. If the patients receive invasive mechanical ventilation after being sedated, the use of sedative drugs may be associated with aspiration pneumonia, shock, or hypoxemia^31^. However, if HRCT is performed as early as possible as in this study, unexpected adverse reactions may be avoided. Mortality was only rarely found in the transport process of patients with early ALI in previous reports^18,32,33^. No mortality was found in the transport process among these patients in the present study.

In conclusion, our findings support the use of HRCT for the identification of of [iSN-aPTB], trial of antibacterial treatment and isolation polices. Meanwhile, when in the unavailable area of the HRCT model (NPV, 99.1%; in derivation cohort), the [CXR + Hypoalbuminemia] model (NPV, 93.4%; in derivation cohort) has the ability to correctly classify the [non-aPTB-PD]. Thus, this simple model may avoid overuse of the isolation room. Finally, owing to the high likelihood ratio (for derivation cohort, 71), the post-test probability in the lower prevalence (1%) is 41.5%. Therefore, this model may be useful in this area^34^ and may be an alternative choice or have an auxiliary role with bronchoscopy among the iSN patients, especially when the sputum-smear status is (−, −, −). Consequently, there is still a need to wait for culture results if a sputum-smear status is (−, −, −) although additional studies are needed to further address this issue.

## Methods

### Ethical considerations

The present study was performed in accordance with the Declaration of Helsinki. The ethics committee of Ping-Tung Christian Hospital approved the study protocol (IRB-268A) and all patients receiving HRCT provided signed informed consent to participate in the study.

### Study design and patient population

This retrospective study enrolled 333 patients with pulmonary diseases having ALI and receiving CXR examination from March 2005 to December 2008 at Pingtung Christian Hospital and its branches. All patients were admitted to the intensive care unit (ICU) where PTB is at high risk of hospital nosocomial infection. All 333 patients had ≥1 immune impairment associated with immunocompromising factors (eg, age >65 years, COPD, DM, uremia, liver cirrhosis, steroid use, lymphopenia, neutropenia, lower Body Mass Index, Hypoalbuminemia)^22^, which predispose patients to aPTB. Therefore, aPTB was suspected among these patients based on signs and symptoms at the time of clinical presentation (Table 1). Consequently, these 333 patients received 3 sets of acid fast bacilli examination, TB culture and CXR. Patients were excluded if they did not have HRCT (n = 15) and if the author was not on duty at the time of examination. Patients were also excluded if the results were indicative of artifacts such as procedural artifacts (N = 5), including motion artifact of the heart (n = 1), respiratory artifacts (n = 3), or pulsation or star artifacts (n = 1). Patients were also excluded if the initial sputum smear was positive (iSP) for AFB (n = 54) aPTB [iSP-aPTB]. Cardiac motion artifacts sometimes interfered with the accurate evaluation of the extent of areas with ground-glass attenuation due to predominant distribution in the lower lobe, including pericardiac areas^35^. The remaining patients (n = 259) with initial smear-negative pulmonary diseases (iSN-PD) were included (Supplementary Fig. S1). After confirmation of diagnosis, these 259 patients were divided into two groups: group 1: G1 [iSN-aPTB], n = 26: [(−, +, +), n = 4; (−, −, +), n = 8; (−, +, −), n = 6; (−, −, −), n = 8]; group 2: G2 [non-aPTB-PD], (n = 233). We analyzed patients’ clinical manifestations (eg, underlying diseases and clinical symptoms/signs) and CXR/HRCT. Thus, we developed the [HRCT/CXR + clinical manifestations] model for identifying the [iSP-aPTB] and correctly classifying [non-aPTB-PD].

A diagnosis of ALI included the following: acute hypoxia with a ratio of partial pressure of arterial oxygen to the fraction of inspired oxygen (PaO_2_: F_I_ O_2_) ≤ 300 mmHg with or without of elevation of arterial PaCO_2_, the presence of bilateral infiltrates seen by CXR, and no evidence of left atrial hypertension^36^.

The management of ALI consisted of oxygen therapy. Of the 259 included patients, 254/259 received HRCT in the ER and 5 out of 259 received the HRCT while admitted to the ICU in the derivation cohort. (Supplementary Table S4). All these patients received HRCT within 24 hours after diagnosis of ALI. Patients were transported from the ER (254 out of 259) or ICU (5 ou of 259) to the CT for HRCT.

#### Validation cohort (all patients having final confirmed diagnosis after admission to the ICU)

Diagnostic models (see Statistical analysis) derived in the derivation phase of the study were tested for diagnostic accuracy in patients who were enrolled from January 2009 to December 2011 with pulmonary diseases having ALI (n = 563). A total of 563 patients had ≥1 immune impairment associated with immunocompromising factors such as in the derivation cohort. All patients received 3 sets of TB smear, culture and CXR. The patients were excluded if they had ALI and the initial sputum smear was positive (iSP) for AFB (n = 161) aPTB or the patients without the HRCT (n = 20), or if the author was not on duty at the time of examination. The artifacts of the procedure (n = 10) included cohort motion artifacts of the heart (n = 2), respiratory artifacts (n = 5), pulsation or star artifacts (n = 3)^25^; the remaining 372 [iSN-PD] patients were enrolled into the validation cohort. (Supplementary Fig. S2) While these patients waited for the 2^nd^ or 3^rd^ smear-positive sputum, HRCT was performed within 24 hours. Of the included 372 patients, 366 /372 received the HRCT at ER and 6 out of 372 received the HRCT during ICU. (Supplementary Table S4). All patients received HRCT within 24 hours after diagnosis of ALI. We transported patients from the ER (366 out of 372) or ICU (6 out of 372) to the CT for HRCT. We tested the ability of the model [HRCT/CXR + underlying diseases and clinical symptom/sign] for detecting the [iSN-aPTB] and classifying the [non-aPTB-PD] among the enrolled patients.

### APACHE II score and ALI score

The Acute Physiology, Age, and Chronic Health Evaluation (APACHE) II score is a prognostic scoring system for disease severity of critically ill patients in the ICU^37^. The 12 variables used to calculate the APACHE II score include: blood pressure, heart rate, respiratory rate, body temperature, serum level of sodium, serum level of potassium, serum level of creatinine, arterial pH, alveolar-arterial oxygen gradient, hematocrit, white blood cell count, and score on the Glasgow Coma Scale^37^. The APACHE II scores were calculated from the sum of weighted points representing the extent of physiological derangements (acute physiology score) and points based on the patient’s age and chronic illness. The worst acute physiology scores recorded during the initial 24 hours of the ICU stay were used. The APACHE II score for each patient was determined at the time of admission by ICU nurses trained in using this instrument.

The ALI scores included CXR evaluated for alveolar consolidation, PaO_2_: F_I_ O_2_, positive end expiratory pressure (PEEP) level if ventilated, and respiratory compliance if known^38^.

### Polymerase Chain Reaction (PCR)

Roche Amplicor Mycobacterium test (Amplicor MTB; Roche Diagnostic Systems, Somerville, NJ, USA) for detecting tuberculosis bacilli in smear-positive specimens. When the first set sputum was negative, results from a 2nd test from spontaneous sputum was collected. If the spontaneous sputum was unavailable after 3 days, the sputum was obtained during bronchoscopy or from the invasive airway (eg, intubation airway). Time to the results of PCR or TB culture are revealed in Table 1.

### Definition of Smear-positive aPTB (AFB +)

The aPTB (AFB + ) is defined as: 1) ≥2 sputum smear positive Acid Fast Bacilli [(+, −, +)(+, +,−) (−, +, +)(+, +, +)]; or 2) 1 sputum smear positive for AFB Smear-positive + chest X-ray consistent with aPTB as determined by the treating medical officer [(+, −, −)(−, + −, −)(−, −, +)]; or 3) 1 sputum specimen positive for AFB [(+, −, −)(−, +, −, −)(−, −, +)] and culture positive for AFB, according to our previous study^16^.

### Hematological and biochemical data

Blood cultures and appropriate culture specimens from the infection were obtained. Hematological and biochemical data also were collected systematically on the day of ER admission. The white blood count, lymphocyte count, neutrophil count, and hemoglobin were measured using standard commercial kits (Sysmex XE-5000, Sysmex Corporation, Japan) and performed using SYSMEX 2100. Ante cibum (AC), blood urea nitrogen (BUN), Creatinine (Cr), albumin, aspartate transaminase (GOT); alanine transaminase (GPT) concentrations were measured for all subjects using standard methods performed on the Uni-Cel DxC 800 analyzer (Beckman Coulter Inc., USA).

### Arterial blood gas analysis

Blood samples were drawn, placed on ice and immediately transmitted to the laboratory for analysis with a blood gas analyzer (pHOx plus Blood Gas Analyzer, Nova Biomedical, Waltham MA, USA). The accuracy of the analyzer was validated daily according to standard quality assurance protocols.

### Evaluation of chest X-ray and high resolution computed tomography

CXR (anteroposterior, posteroanterior, laterally) and HRCT images of thoracic lesions were performed at admission and were retrospectively evaluated within 3 days (1 day) of image collection for each patient in derivation (validation) cohort. Both CXR and HRCT were evaluated by three radiologists, all of whom had >15 years of experience, and two chest physicians, one of whom had >20 years of experience and the other had five years of experience. Both types of images were evaluated simultaneously such that the radiologist could refer to and compare them. Final decisions on the imaging results were reached by consensus within 24 hours. The inter-observer and intra-observer agreement for the CXR and HRCT showed high Cohen’s kappa values of >0.85.

#### CXR

The pattern of parenchymal lesions seen on CXR was classified into having air-space consolidation/patch, small nodules/reticular lesions, and/or cavitation. The lung was divided into 4 regions for analysis. Lesions were designated as being in the right/left upper lung field and right/left lower lung field, as previously described^39^.

#### High resolution computed tomography

Figures 1, 2 and Supplementary Fig. S3. High resolution CT scans were performed using a 64-MDCT scanner (Brilliance, Philips Medical Systems, Cleveland OH, USA) set to 0.625 mm collimation, 100–120 kV, 250 mAs, a table speed of 57.5 mm/sec, a rotation time of 0.75 sec, and a pitch of 1.07. The images were acquired during a single breath-hold lasting five to eight seconds, with nasal cannula (n = 11) or mask with oxygen before the invasive ventilator support (n = 195) or while trying the mask on the non–invasive ventilator set (n = 48) in the early stage of ALI^18^ among patients with spontaneous breathing. In the patients without spontaneous breathing, the images were acquired during end phase of inspiration (n = 5) after portable invasive ventilator set was on^40,41^. Respiratory motion is greatest at the lung bases and scanning in a caudal-to-cranial direction may help to minimize respiratory artifacts because scans through the lung bases are acquired when the patient is holding his or her breath. However, methods that render respiratory motion artifacts are uncommon in most patients. The images were reconstructed with a 1-mm slice thickness in the axial plane (no gap) and in the coronal plane (5-mm apart) using a high spatial-frequency algorithm, and subsequently sent to picture archiving and communications system (PACS) for review. All thin-section MDCT images were displayed on a monitor at the pulmonary window level setting (level, −600 HU; width, 1200 HU).

For HRCT analysis, the lung was divided into 12 segments (See Table 2 footnote). The HRCT morphology, including consolidation (lobar, segment, subsegment, lobular), cavitation, clusters nodules/mass, branching centrilobular nodular lesions (ie, tree-in-bud pattern), centrilobular nodules, reticular nodules, interlobular thickening, ground-glass opacity and pleural effusion were analyzed. The peripheral distribution of consolidation, cavitation, and clusters nodules/mass distribution were also analyzed. Findings were reached by consensus. The kappa values of CXR and HRCT morphology reading results were >0.85.

### Statistical analysis

Data are presented as mean ± standard deviation (SD) for continuous variables and numbers with percentages (%) for categorical variables. Two-sample t-test was used to compare the differences between groups for continuous data. Pearson’s Chi-square test or Fisher’s exact test was used to compare the differences in distribution of the categorical data between groups. Multiple logistic regression analysis was performed to indicate the predictors of the subjects with [iSN-aPTB]. The estimated beta (β) with standard error and odds ratio (OR) with 95% confidence interval (CI) were determined. A relative score used in the prediction model was given for the different independent variables by using the estimated β as a base as described in the section of the diagnostic model and evaluation of diagnostic accuracy. All statistical analyses were considered significant at P < 0.05. We also tested the ability of this model using post-test probability in these two phases and a given prevalence. Statistical analyses were performed using SPSS 15.0 statistics software (SPSS Inc., Chicago IL, USA).

## Supplementary information

Supplementary information

## Data Availability

All data presented in the manuscript are readily available.
