# Supplementary material for: Predictors of Initial Smear-Negative Active Pulmonary Tuberculosis with Acute Early Stage Lung Injury by High-Resolution Computed Tomography and Clinical Manifestations: An Auxiliary Model in Critical Patients
Source: Sci Rep. 2019 Mar 14;9:4527. doi: 10.1038/s41598-019-40799-w (PMC6418143; doi:10.1038/s41598-019-40799-w)

Predictors of Initial Smear-Negative Active Pulmonary Tuberculosis with Acute Early Stage Lung Injury by High-Resolution Computed Tomography and Clinical Manifestations: An auxiliary model in critical patients

Jun-Jun Yeh

Supplementary Table S1. The relative score of CXR (including R1, R2, and R3), HRCT, and CXR plus hypoalbuminemia in the G1(G3), G2(G4) populations.

	G1 (n=26)	G2 (n=233)	G3 (n=31)	G4 (n=341)
**CXR**				
**Relative score of R1**				
0	8	127	4	152
1	7	86	19	153
2	11	20	8	36
**Relative score of R2**				
0	8	129	5	151
1	7	84	18	154
2	11	20	8	36
**Relative score of R3**				
0	8	128	5	152
1	7	85	18	153
2	11	20	8	36
**HRCT score**				
0	1	214	2	330
1	0	11	0	7
2	1	5	0	2
3	24	3	29	2
**CXR score + Hypoalbuminemia**				
0	7	123	4	146
1	8	90	19	159
2	11	20	8	36

Data were summarized as number of patients in G1, G2, G3, and G4.

R1, Radiologist 1; R2, Radiologist 2; R3, Radiologist 3

Supplementary Table S2. Summary of patients in R1 vs. R2 of HRCT and CXR in the derivation cohort

		Relative score (R1)			
		0	1	2	3
HRCT					
Relative score	0	211	1	0	0
(R2)	1	3	11	0	0
	2	0	0	6	2
	3	0	0	1	24
CXR					
Relative score	0	130	7	0	-
(R2)	1	5	86	0	-
	2	0	0	31	-
	3	-	-	-	-

Data were summarized as number of patients.

R1, Radiologist 1; R2, Radiolgists 2; R3, Radiologist 3

Supplementary Table S3. The sensitivity, specificity, positive predictive value, and negative predictive value of HRCT and CXR plus Hypoalbuminemia models in the new validation cohort※ (N=155).

Predictive results	CXR + Hypoalbuminemia score		HRCT score§	
	Predicted iSN-aPTB (n=12) ≥ 2	Predicted [non-aPTB-PD] (n=143) < 2	Predicted iSN-aPTB (n=12) ≥ 3	Predicted [non-aPTB-PD] (n=143) < 3
G5 (n=12) **	5	7	11	1
G6 (n=143)	7	136	1	142
**Predictive terms**				
Sensitivity	41.6% (5/12)		91.6% (11/12)	
Specificity	95.1% (136/143)		99.3% (142/143)	
False positive rate	4.9% (7/143)		0.7% (1/143)	
False negative rate	58.4% (7/12)		8.4% (2/31)	
Positive predictive value	41.6% (5/12)		91.6% (11/12)	
Negative predictive value	95.1% (136/143)		99.3% (124/143)	

CXR, chest X-ray; False positive rate=1-specificity; False negative rate=1-sensitivity.
G5 (N=12), initial smear-negative active pulmonary tuberculosis (iSN-aPTB);
G6=non-aPTB pulmonary diseases [non-aPTB-PD]

★ Streptococcus pneumoniae (by blood culture and urinary antigen)

* Includes aPTB with bacteria★ (n=1, grouped the [aPTB concomitant with Streptococcus pneumoniae] as [iSN-aPTB,G5]) **

G6 including (n=143): collagen vascular disease (n=2), lung cancer or metastatic cancer to lung (n=10),smear-negative aPTB (n=9),non-tuberculosis mycobacterium (n=5), cryptococcosis (n=1), aspergillosis (n=1), pneumonia (n=102), actinomycosis (n=0),other lung disease (n=13) such as pulmonary edema(n=12) sarcoidosis (n=0),

hypersensitivity pneumonitis (n=1).
New validation cohort prevalence=12/155=7.7%; pre-test odd ratio 7.7/[100-7.7]=0.083; likelihood ratio=91.6/0.7=130; post-test odd ratio=0.083 x 130=10.79; post-test probability=10.79/[10.79+1]=91.5%.
Given a low prevalence =0.1% ; pre-test odd ratio 0.1/[100-0.1]=0.001; likelihood ratio=91.6/0.7=130; post-test odd ratio=0.001 x 130=0.13; post-test probability=0.13/[0.13+1]=56.5%>5% ,it is the optimal level of cut-off threshold for isolation as described previously
§Patients (including [iSN-aPTB concomitant with Streptococcus pneumoniae]) were isolated and received anti-TB therapeutic drug if HRCT total score was ≥ 3. After the confirmed diagnosis of these 155 patients, the model was tested in the **new validation cohort.**

Supplementary Table S4. Derivation cohort (N=259) and Validation (N=372) cohort received HRCT with Nasal/Mask or BiPAP at ER and invasive mechanical ventilator during ICU in early stage ALI

ALL patients received CXR at ER (N=631)	**Derivation Cohort**		**Validation Cohort**	
	(n=259)		(n=372)	
HRCT(Before ICU)※	ER	ICU	ER	ICU
Nasal/ Mask	195+48+(5)/259 ※		196+154+(6)/372 ※	
BiPAP	11 /259		16/372	
Invasive Ventilator				
HRCT(after ICU)∗				
Nasal/Mask		(48/259)		(154/372)
BiPAP		(11/259)		(16/372)
Invasive Ventilator		(195)+5∗ /259-		(196)+6∗ /372

ER: Emergence Room ICU: intensive care unit

On Oxygen nasal cannula/Mask
BiPAP (Bi-Level Positive Air Pressure, BIPAP® FOCUS™ N, Respironics, Philips),
Invasive Mechanical Ventilation

※ Received HRCT at ER(before ICU) except (5)/259 in derivation cohort and (6)/372 in validation cohort

∗ Received HRCT after invasive mechanical ventilation during ICU(after ICU), these 5/259 patients in derivation cohort and 6/372 patients in validation cohort with nasal/mask at ER

Supplementary Table S5. The actual timing of artery blood gas (P/F ratio<300 mmHg), bilateral infiltration in Chest-x-ray and HRCT

**Derivation cohort (time to get data and interpretation, n=259, all with 24 h)**	
**Procedure**	**Time (hour)**
Artery blood gas	1.37±0.42
Chest- x-ray	1.77±0.77
High-resolution computer tomography	7.39±4.69

**Validation cohort (time to data and interpretation, n=372, all with 24 h)**	
**Procedure**	**Time (hour)**
Artery blood gas	1.53± 0.52
Chest- x-ray	2.06± 0. 82
High-resolution computer tomography	8.65± 5.26

Supplementary Figure S1 Flow chart of the derivation cohort. Group1 (G1)=initial smear-negative and finally active pulmonary tuberculosis [iSN-aPTB]; Group2 (G2)=non-aPTB pulmonary diseases [non-aPTB-PD].

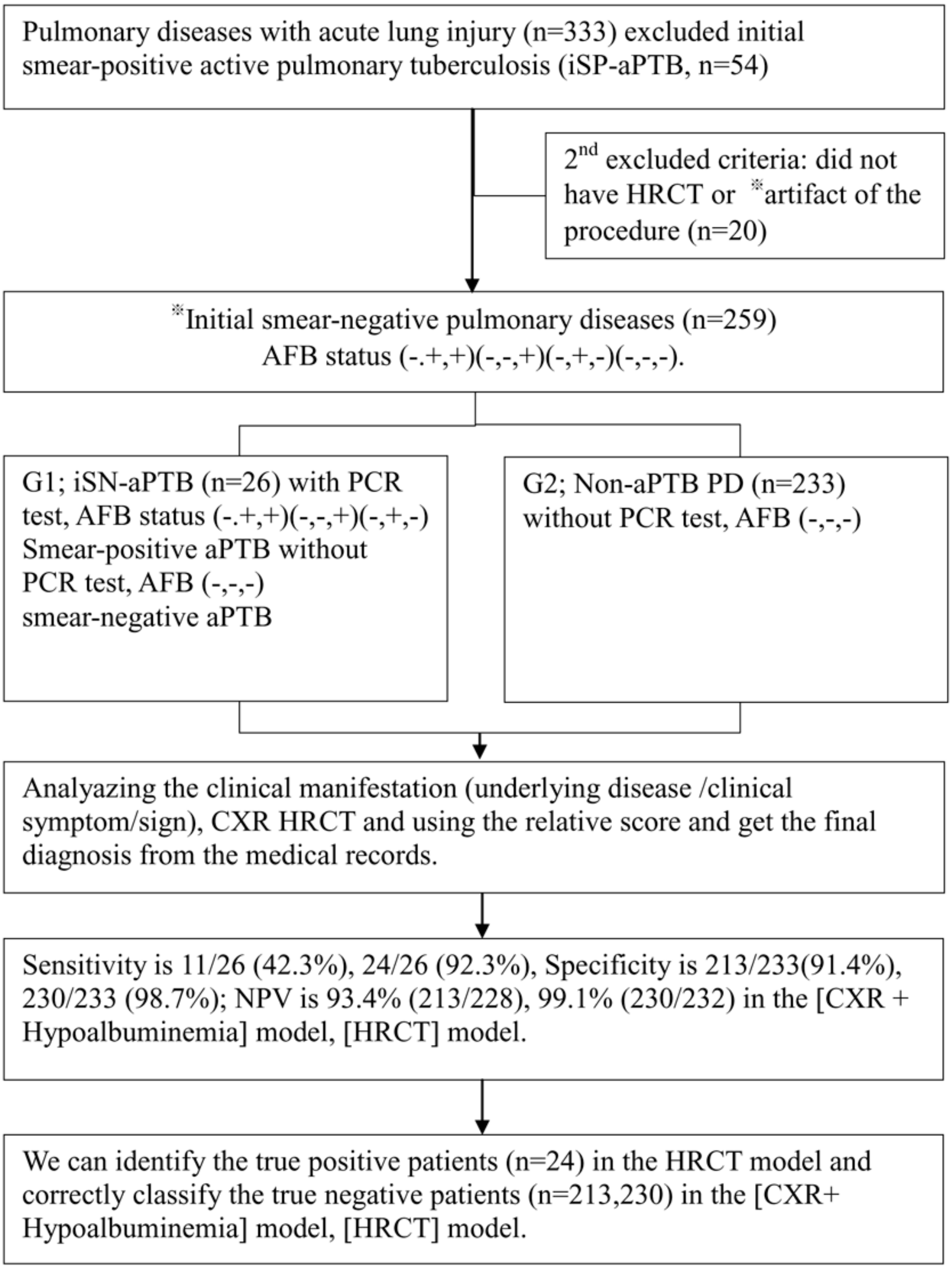

※All the patients having the informed consent of the HRCT when receiving the HRCT at the same time

Supplementary Figure S2 Flow chart of the validation cohort. Group3 (G3)=initial smear-negative active pulmonary tuberculosis (iSN-aPTB); Group G4 (G4)=non-aPTB pulmonary diseases [non-aPTB-PD]

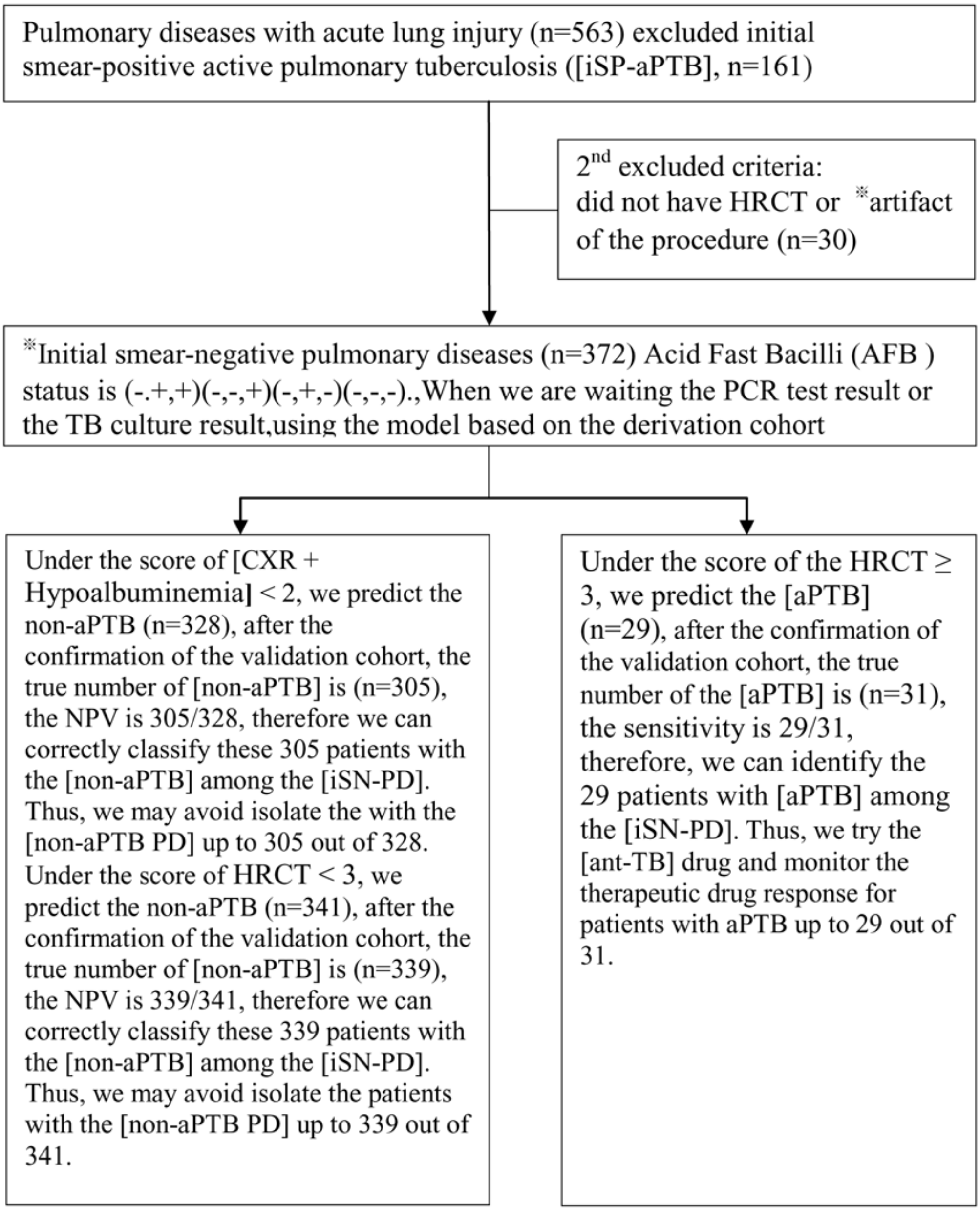

※All the patients having the informed consent of the HRCT , medical records when receiving the HRCT at the same time

Supplementary Figure S3 Presentation of a 74-year-female [aPTB +pneumococcus pneumonia] with acute lung injury, presenting as dyspnea (score=0) in HRCT. The coronal section of HRCT shows consolidation in s6 of right lower lobe (right black arrowhead) (score=1)] and clusters nodules/mass in s1+2 of left supper lobe (score=2) ; black arrow indicates the major fissure (total score =3).

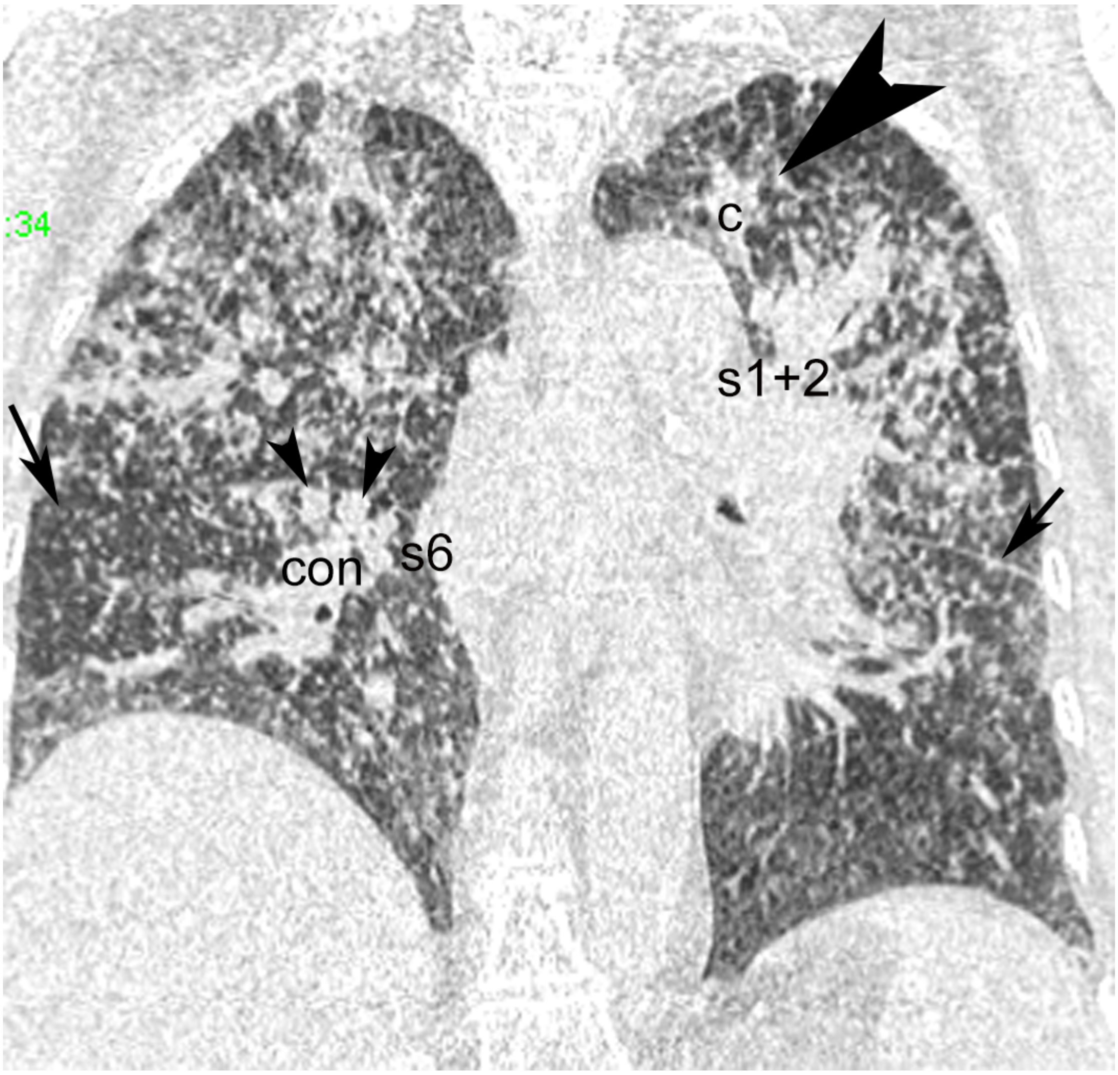

Supplement: Supplementary file 1 — Supplementary information [file 41598_2019_40799_MOESM1_ESM.pdf]
